# Correlation between the results of cultures and the molecular BIOFIRE® joint infection panel in a cohort of pediatric patients with bone and joint infections in Bogotá, Colombia

**DOI:** 10.3389/fped.2024.1359736

**Published:** 2024-04-24

**Authors:** Germán Camacho-Moreno, Enrique Vergara-Amador, Tomás Martínez-Villegas, Yefry Aragón-Joya, Luz Romero-Cardozo, Francisco Lores-Garcia, Vivian Marcela Moreno, Aura Lucia Leal-Castro

**Affiliations:** ^1^ HOMI, Fundación Hospital Pediátrico la Misericordia, Bogotá, Colombia; ^2^ Departamento de Pediatria, Facultad de Medicina, Universidad Nacional de Colombia, Bogotá, Colombia; ^3^ Unidad de Ortopedia, Departamento de Cirugía, Facultad de Medicina, Universidad Nacional de Colombia, Bogotá, Colombia; ^4^ Departamento de Microbiología, Facultad de Medicina, Universidad Nacional de Colombia, Bogotá, Colombia

**Keywords:** bone and joint infection, correlation, children, BIOFIRE® joint infection panel, microbiological cultures, *Staphylococcus aureus*, *Kingella kingae*, *Streptococcus pyogenes*

## Abstract

**Introduction:**

Bone and Joint Infections (BJI) have high morbidity. Methicillin resistant *Staphylococcus aureus* (MRSA) has increased. Culture-based diagnosis has difficult to recovering fastidious bacteria and detecting polymicrobial infections, molecular methods offer a promising improvement for the diagnosis of BJI with reduced time to result. The aim of the study was to determine the correlation between culture results and the Biofire joint infection panel (BJIP) in a cohort of pediatric patients with BJI.

**Materials and methods:**

Descriptive study. Patients admitted with probable o confirmed BJI between July 1, 2019 and February 28, 2021 at HOMI. Blood cultures, synovial and bone fluid samples were taken. Samples were kept at −70 °C. On September 2022, the panel was performed.

**Results:**

32 patients were included**.** The average age was 83m (RIQ: 32–145). 23 (71.8%) patients had a positive culture. The most frequent microorganism were *S. aureus* 19 (83%), 11/19 (57.9%) Staphylococci isolates were MRSA. 24/32 (75%) were positive by panel, 20 positive detections were concordant with culture, there were 6 additional isolates by panel (2 *S. aureus*, 2 *S. pyogenes*, 1 *K. kingae* and 1 *C. albicans*), three microorganisms were isolated in culture but not in the panel. (2 *S. aureus* and 1 *S. agalactiae*). Two patients with coinfection were detected. All MRSA were detected by culture and panel. In 26 (81.3%) patients the etiology was documented by any method.

**Conclusion:**

These results showed a moderate level of agreement between BJIP and culture (*κ* = 0.47). The panel allowed the detection of fastidious bacteria including *K. kingae* and polymicrobial samples. There was a very good level of agreement between the panel and culture for the MRSA detection (*κ* = 1).

## Introduction

Bone and joint infection (BJI) in children has an estimated prevalence of 11 per 100,000 acute osteomyelitis (AO) patients, 6 per 100,000 septic arthritis (SA) patients, and 2 per 100,000 combination (AO + SA) patients. BJI can occur at any age but is more frequent in infants and adolescents. Most patients require hospital management, generating significant costs for health systems (1).

The predominant microorganisms in BJI are *Staphylococcus aureus*, *Streptococcus pyogenes*, *Streptococcus pneumoniae* and *Kingella Kingae* (2–4). In terms of pathophysiology, bacteremic dissemination is more frequent in children than in adults, which typically occurs by contiguity (4). Signs and symptoms vary with age, the most frequent being pain in the affected limb or joint, increased joint or periarticular volume, erythema, and fever (5). Treatment often requires surgical drainage and antibiotics that cover the most frequent microorganisms, whose durations range between 21 and 42 days, depending on the isolated microorganism and the presence or absence of complications (3, 6).

Regarding the performance of diagnostic tests, the positivity of blood cultures is 30%–40%, and that of fluid cultures is between 60% and 70% (5, 6). Using only those methods of bacterial identification, it is not possible to know the etiology of 30%–40% of BJI cases (5, 6). The use of molecular biology methods has increased the recovery rate of fastidious microorganisms such as *Kingella kingae* (3, 7, 8).

Multiplex molecular panels speed up the identification of pathogens and identify microorganisms that are difficult to grow in cultures, in addition to providing information about the resistance genes that they possess. The present study aims to describe the clinical characteristics of the patients and to correlate the results of the BIOFIRE® joint infection panel with the results of microbiological cultures, and to quantify the prevalence of pathogens that are difficult to isolate in culture, such as *Kingella kingae*, in a cohort of children with BJI treated in a reference hospital in the city of Bogotá, Colombia.

## Materials and methods

This is a prospective descriptive study of patients admitted with BJI between July 1, 2019 and February 28, 2021 at HOMI, Fundación Hospital Pediátrico La Misericordia, Bogotá, Colombia. The medical status was recorded, and blood cultures, bone fluid cultures, and synovial fluid samples were taken. Only patients in whom a sample of joint fluid or bone pus was taken were selected. The samples were stored at −70  °C in the Microbiology Laboratory of the Universidad Nacional de Colombia for their conservation. In September 2022, research use only version BIOFIRE® joint infection panel was performed on synovial fluid and bone fluid.

The BIOFIRE® joint infection panel allows, in a single sample of synovial fluid, the simultaneous analysis of several aerobic and anaerobic bacteria and yeasts that cause BJI, as well as genetic markers associated with resistance to antibiotics, through PCR. The final version of the panel was approved by the FDA for use on fresh (<7 days at 2–8  °C after sampling), synovial fluids samples only. The present study was designed and started with a research use only version of the panel before FDA approval and includes both synovial fluid samples and bone fluid; samples had been frozen at −70  °C. The final version of the panel contains the same molecular targets as the one used in this study. Using the panel on bone fluid or frozen samples would be considered off-label with the FDA-approved version of the panel. The results were not used to change the treatment of the patients; permission was requested from INVIMA to carry out the study.

Table 1 lists the 31 pathogens and the eight genetic resistance markers covered by the panel used in this work.

**Table 1:** BIOFIRE® joint infection panel identification.

Gram-positive bacteria	Gram-negative bacteria
• *Staphylococcus aureus* • *Staphylococcus lugdunensis* • *Streptococcus* spp. ○ *Streptococcus pyogenes* ○ *Streptococcus agalactiae* ○ *Streptococcus pneumoniae* • *Enterococcus faecalis* • *Enterococcus faecium* • *Anaerococcus previti/vaginalis* • *Peptoniphilus* • *Clostridium perfringens* • *Cutibacterium avidum/granulosum* • *Finegoldia magna* • *Parvimonas micra* • *Peptostreptococcus anaerobius*	• *Escherichia coli* • *Proteus* spp. • *Citrobacter* • *Enterobacter cloacae complex* • *Klebsiella aerogenes* • *Klebsiella pneumoniae group* • *Morganella morganii* • *Serratia marcescens* • *Salmonella* spp. • *Kingella kingae* • *Pseudomonas aeruginosa* • *Haemophilus influenzae* • *Neisseria gonorrhoeae* • *Bacteroides fragilis*
Yeast	Antimicrobial resistance markers
• *Candida* ○ *Candida albicans*	• *mecA* + MREJ • Van A/B • CTX-M • KPC, NDM, OXA-48-like, VIM, IMP

## Results

Thirty-eight patients with suspected BJI were included, six were excluded for having etiologies other than BJI as the final diagnosis (three with juvenile idiopathic arthritis, one with cellulitis, one with myositis without BJI, and one with Langerhans cell histiocytosis). Finally, 32 patients with probable or confirmed BJI were included, of whom 14 (43.7%) had septic arthritis, 11 (34.4%) had osteomyelitis, and 7 (21.9%) had both (AO + SA). Of the patients with joint involvement, three had involvement in more than one joint. The most frequently involved joints were knee, 9 (28.1%); hip, 7 (21.9%); elbow, 3 (9.4%); ankle, 3 (9.4%); and shoulder, 2 (6.3%). Of the patients with bone involvement, three had multifocal involvement, and the most frequently affected bones were the femur, 10 (47.6%); humerus, 3 (14.2%); fibula, 2 (9.5%); tibia, 2 (9; 5%); clavicle, 2 (9.5%); ulna, 1 (4.8%); and talus, 1 (4.8%).

### Demographic and clinical results

Seventeen (53%) of the patients were male. The median age was 83 months (RIQ: 32–145 months). The most common age range was 10–15 years (14 patients), followed by under 2 years (8 patients) (Table 2). The most frequent symptoms were joint pain (100%), fever (75%), edema and erythema (74%), and lameness (69%) (Supplementary Table S1). The median time between the onset of symptoms and the consultation was 3 days (RIQ: 2–7 days). The median duration of fever was 4 days (RIQ: 1–7). Fifteen (47%) of the patients had some comorbidity: 4 with musculoskeletal disorders (2 with chronic osteomyelitis, 1 hip dysplasia, 1 history of fracture), 3 with neurological diseases (2 Epilepsy and 1 Lesch-Nyhan Syndrome), 2 with rheumatological diseases (systemic lupus erythematosus), 2 with respiratory diseases (Asthma), 1 with cancer (acute lymphoblastic leukemia), 1 with coagulation disorders (Hemophilia B), 1 with endocrine disorders (Hypothyroidism) and 1 with kidney disorders (Hydronephrosis grade 1). Ten (31%) patients received antibiotics before admission, oxacillin and clindamycin being the most widely used. The median length of hospital stay was 13 days (RIQ: 9–21 days), all patients underwent surgical procedures, 20 (63%) needed two or more surgeries, and 4 (12.5%) of the patients were admitted to the pediatric intensive care unit (PICU), with an median stay of 2 days (RIQ: 1–9). There were no deaths (Table 2).

**Table 2:** General demographics and clinical outcomes.

Variable	*n*	%
Total patients	32	100
Age		
6–12 months	2	6
1–2 years	6	19
3–5 years	7	22
6–9 years	3	9
10–12 years	7	22
13–15 years	7	22
Sex		
Male	17	53
Female	15	47
Diagnosis		
Septic arthritis	15	47
Osteomyelitis	12	37
Both (SA + AO)	5	16
Antibiotic before admission	10	31
Requirement for surgical intervention	32	100
Performance of 2 or more surgical interventions	20	63
Patients by number of surgeries performed		
1 time	12	38
2 times	7	22
3 times	5	15
4 times	7	22
5 times	0	0
6 times	1	3
Admission to PICU	4	12.5
Deaths	0	0

Supplementary Table S2 shows the behavior of the blood count and the acute-phase reactants. After treatment, leukocytes and neutrophils decreased, C-reactive protein decreased, and platelets and erythrocyte sedimentation decreased. C-reactive protein took an median of 6 days (RIQ: 3–10) to decrease 50%.

Plain radiography was performed in 30 (94%) of the patients, ultrasound in 27 (84%), and nuclear magnetic resonance in 22 (68.8%). The most frequent findings in the images are described in Supplementary Table S3.

### Microbiological results

**Figure 1:**
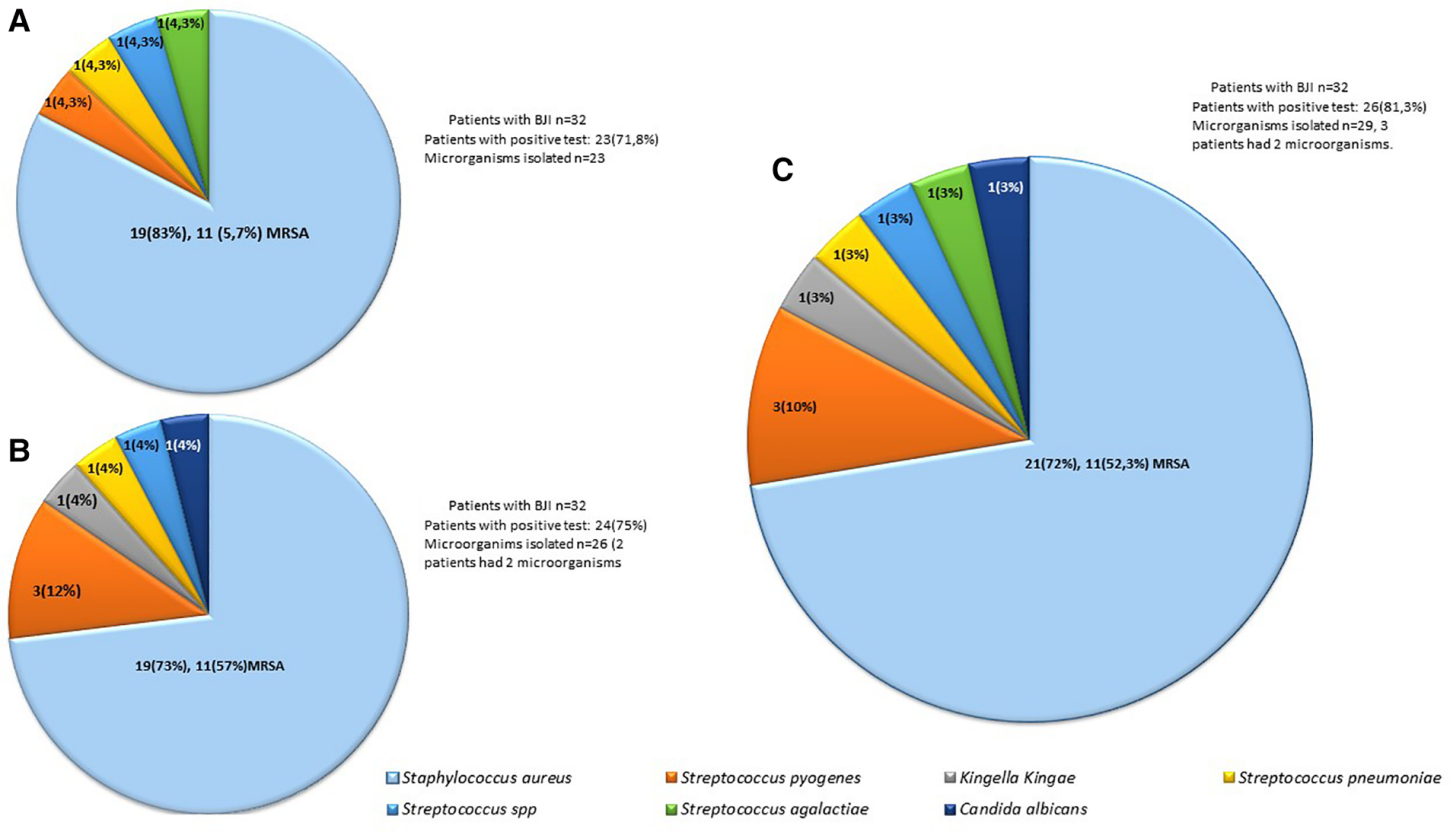
Frequency of microorganisms identified by culture (**A**), the BIOFIRE® joint infection panel (**B**) or both (**C**).

Fluid cultures were performed for all 32 patients, and blood cultures were performed for 25 (78.1%). A microorganism was isolated in 23 (71.8%) of the fluid cultures and in 11 (44%) of the blood cultures. In none of the cultures was more than one microorganism reported, and no patient with a negative fluid culture had a positive blood culture. The microorganism that was most documented in blood culture was *S. aureus* 10/11 (90.9%). The positivity rate of the cultures (fluid culture + blood culture) was 71.8%. The most frequently isolated microorganism in the overall cultures was *S. aureus*, representing 83%, of which 57.8% were resistant to methicillin. Isolation of *Streptococcus pyogenes*, *Streptococcus pneumoniae*, *Streptococcus dysgalactiae*, and *Streptococcus agalactiae* was also documented, each in 4.3% of the cases (Figure 1A).

A multiplex molecular panel was performed on the 38 samples of patients with suspected BJI. In the 6 patients in whom BJI was ruled out based on clinical presentation, the result of the joint fluid panel was negative. Of the 32 patients with probable or confirmed BJI, in 24 (75%) of the patients, at least one microbiological isolation was detected; in two of them, two microbiological isolates were detected (in one patient *S. aureus* without resistance genes and *Streptococcus* spp., and in another *S. aureus* without resistance genes and *Streptococcus pyogenes*). In 25/32 (78.1%) patients, the culture result was concordant with the panel result, indicating a moderate level of agreement (*κ* = 0.47). Of the 26 microbiological isolates documented in the panel, the most frequent was *S. aureus* (73%), 57% of which had the *mecA* gene. The second most common microorganism was *Streptococcus pyogenes* (12%), followed by *Kingella kingae*, *Streptococcus pneumoniae*, *Streptococcus* spp., and *Candida albicans* (4% each) (Figure 1B). When analyzing by type of sample, 21 joint fluid samples were processed, 13 (62%) of which were positive, eight for *S. aureus* (four methicillin-resistant), one with coinfection with *Streptococcus* spp., two for *Streptococcus pyogenes*, one for *Kingella kingae*, one for *Streptococcus pyogenes* and one for *Candida albicans*. 11 bone pus were processed, with 100% positivity, all positive for *S. aureus* (seven methicillin-resistant), one of them with coinfection by methicillin-sensitive *S. aureus* and *Streptococcus pyogenes*.

When analyzing the patients who had a positive culture and/or panel, we found that a microorganism was identified in 26 (81.3%) of the patients and that 29 microorganisms were isolated; in three patients, two microorganisms were documented, two of them in the molecular panel previously described, and in another immunosuppressed patient, *Streptococcus agalactiae* was documented in culture and *Candida albicans* in the panel*.* The most frequent microorganisms were *S. aureus* at 72% (52.3% methicillin-resistant), followed by *Streptococcus pyogenes* at 10%, *Kingella kingae*, *Streptococcus pneumoniae*, *Streptococcus* spp. (*S. dysgalactiae*), and *Streptococcus agalactiae* (Figure 1).

Of the 3 patients with multifocal involvement, *Staphylococcus aureus* was identified in 2 patients (1 MSSA and 1 MRSA) in cultures and in the molecular test and *Streptococcus pyogenes* in one patient documented only in the molecular test. Of the 10 (31%) patients who previously received antibiotics, 9 had a positives results in the culture and the molecular panel and one had a positive result only in the molecular panel. The previous use of antibiotics was not correlated with a decrease in the detection rate of microorganisms.

**Figure 2:**
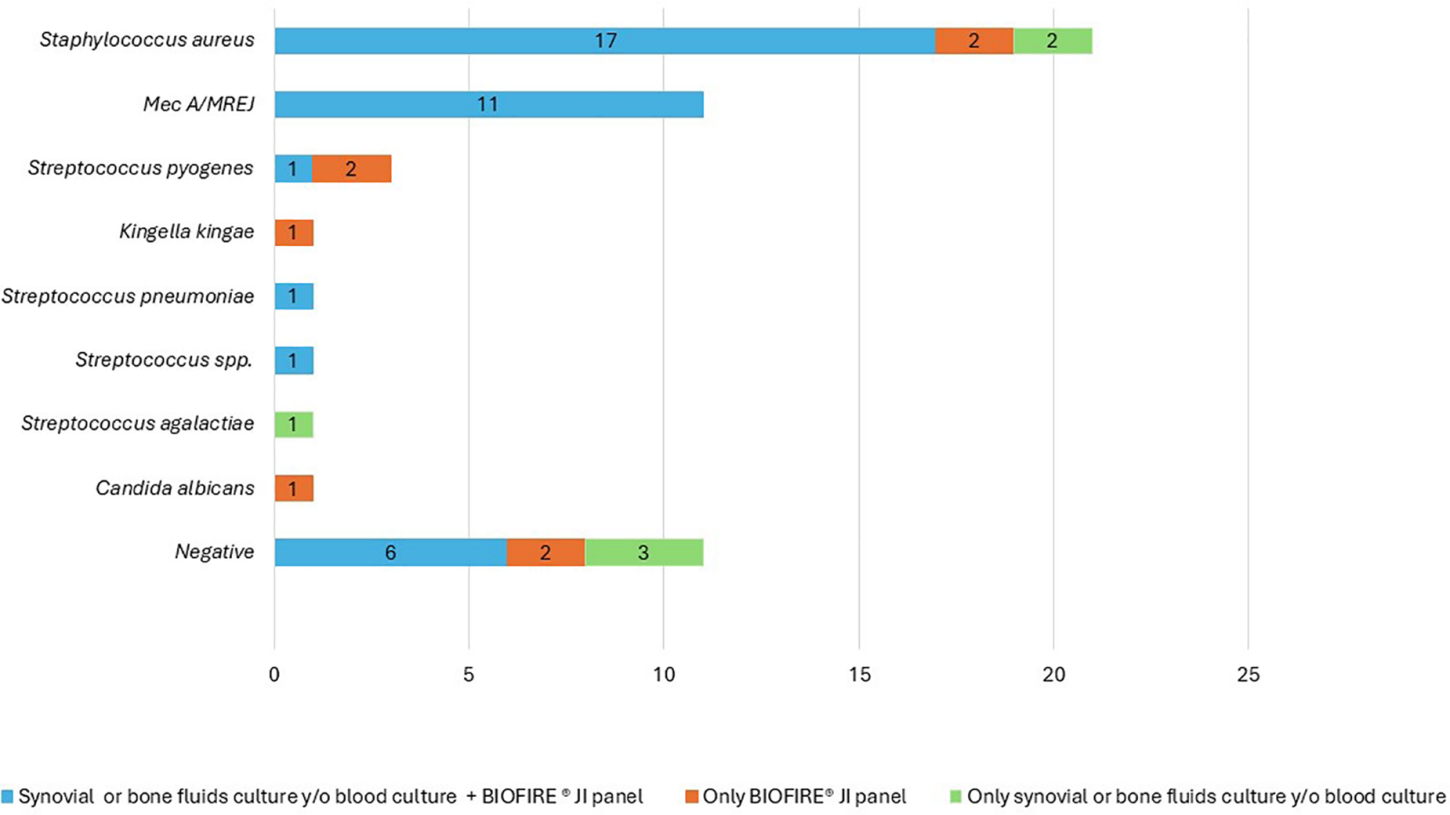
Correlation between microorganisms isolated in synovial and/or bone fluid culture or blood culture and/or BIOFIRE® joint infection panel.

Figure 2 shows the correlation between the result of synovial fluid and/or bone fluid and/or blood cultures and the result of the molecular panel for each of the microorganisms. Seventeen isolates of *S. aureus* were documented both in the blood culture and in the molecular panel. Two isolates were documented in the molecular panel but not in the culture. One patient had *Streptococcus dysgalactiae* in blood culture while the panel detected both *S. aureus* without resistance genes and *Streptococcus* spp. in the panel. Another had negative culture results but a panel in which *S. aureus* without resistance genes and *Streptococcus pyogenes* were identified*.* Two microorganisms were isolated in culture but not in the panel. These two cases were analyzed: The first was a 2-year-old male patient with septic knee arthritis. In a culture out of five, methicillin-sensitive *Staphylococcus aureus* grew, taken from the second wash in an anaerobic bottle. All the cultures of the first wash were negative, and the sample analyzed by the molecular panel was from those first cultures. The second patient was a male patient aged 1 year 9 months with septic arthritis of the right knee + soft tissue infection, which needed four surgical washings. In the first wash, four samples were taken for culture, one of which was positive for methicillin-sensitive *Staphylococcus aureus*. The panel was run on a different sample from the first wash. Of the 21 isolates of *S. aureus*, 11 were methicillin-resistant, and all of them were detected both in the culture and in the molecular test (*κ* = 1).

An isolate of *Streptococcus pyogenes* was documented both in the culture and in the molecular test, and two isolates of this microorganism were documented in the molecular test and not in the culture, one of them with coinfection with *S. aureus*. There were no isolates of *Kingella kingae* in cultures, and isolation of this microorganism was documented in the molecular panel in a girl of 2 years 7 months who consulted for a 3-day picture of pain in the right knee, rhinorrhea, and cough, without fever. This patient had a normal hemogram, elevated CRP, joint edema, x-ray with soft tissue edema, and ultrasound with soft tissue edema and increased joint fluid. A diagnosis of right knee septic arthritis was made, and the patient received empirical management with clindamycin to cover *S. aureus*, which needed four surgical washes. The culture was negative. She evolved to improvement and discharge with trimethoprim sulfamethoxazole. The antibiotic therapy was not modified to a treatment with adequate spectrum against *Kingella kingae* because the panel was conducted in September 2022, 3 years after discharge.

One patient had isolation of *Streptococcus pneumoniae* both in the culture and in the molecular panel, and one patient had isolation of *Streptococcus dysgalactiae* in culture that was identified in the panel as *Streptococcus* spp. In the same patient, the panel additionally identified *S. aureus* without resistance markers, which had not been reported in culture.

One patient had *Streptococcus agalactiae* identified in culture, while the panel detected *Candida albicans*. This was a 14-year-old female patient with a history of systemic lupus erythematosus, neutropenia on admission to NT 900, septic arthritis of the left elbow, and initial management with clindamycin. With the result of the culture, ampicillin therapy was started, with good evolution. In this case, the detection of *Candida albicans* could be a contamination.

Of the nine patients with a negative culture, six had a negative molecular panel and three had a positive molecular panel. In one, *Kingella kingae* was identified; in another, *Streptococcus pyogenes*; and in another, *S. aureus* without resistance genes and *Streptococcus pyogenes*.

### Antibiotic management

The 32 patients received empirical antibiotic management: clindamycin in 24 (75%), vancomycin in 4 (12.5%), cefazolin in 3 (9.4%), and oxacillin in 1 (3.1%). Therapy was changed by reducing the spectrum in 8 (25%) of the patients, and the time between the initial scheme and the definitive scheme (guided by cultures) in these patients was 2.6 days. In 2 (6.25%) patients, it was necessary to increase the spectrum due to resistance to initial management, after an average of 3 days. The panel results were not used to change the treatment of the patients.

## Discussion

BJI is a frequent pathology in pediatrics. In the present study, the distribution of diagnoses was similar to those found in other studies (9–11). The average age was similar to those reported by some studies (10, 12, 13) but older than that in other studies where infants predominated (9). The time between the onset of symptoms and the consultation was longer than reported (5, 9). The clinical characteristics of the patients, the distribution of the affected bones and joints, and the laboratory and radiological findings are similar to those found in other studies (5, 9–13). Hospital stay and clinical outcomes are also similar (9–11).

As for the microbiological findings, the most frequent microorganisms isolated in culture were *S. aureus*, *Streptococcus pyogenes*, and *Streptococcus pneumoniae*, similar to those found in other studies (2, 4, 5). Blood cultures were performed in 25/32 (78.1%) patients. The proportion of positivity of blood cultures, 11/25 (44%), was similar to that reported in other studies (6).

All patients had cultures performed at the time of surgery (bone, joint fluid, or discharge), 23/32 (71.8%) with a positive result, data similar to that found in other studies (6). No patient with a negative fluid culture had a positive blood culture, and the total positivity of blood culture + fluid culture was 71.8%, similar to that found in the literature (6).

The molecular test was positive in 24/32 patients (75%). In two patients, two microorganisms were found (one with *Streptococcus* spp. and *S. aureus* and another with *S. aureus* and *Streptococcus pyogenes*). The prevalence of methicillin-resistant *S. aureus* was 52.3%, slightly higher than that reported in other studies in Colombia (14, 15). The correlation between the phenotypic profile and the detection of the *mecA* gene was 100%. Other molecular tests which detect methicillin resistance have be reported to impact time to adequate therapy in several types of infection. Mortality in methicillin-sensitive *S. aureus* infections increases with the use of vancomycin (16).

The implementation of the multiplex molecular panels increased the microbiological recovery by 10%. In the present study, the panel detected some microorganisms that were not detected in cultures: two isolates of methicillin-sensitive *S. aureus*, two of *S. pyogenes*, one *Kingella kingae* considered the cause of the infection, and one *Candida albicans* considered contamination, but failed to detect two isolates of *S. aureus* and one isolate of *Streptococcus agalactiae*, these pathogens were detected in other samples from the same patient, taking several samples, including tissue, is the standard for diagnosis by culture and the fact that the panel is negative does not allow us to rule out an infection that could be detectable in another sample. Other studies show that the implementation of molecular biology techniques increases the percentage of microbiological recovery, especially in preschool patients, by increasing the detection, especially of *Kingella kingae* and other pyogenic bacteria (3, 7, 8, 17, 18).

When molecular techniques are used, the isolation of *Kingella kingae* significantly increases, this being the most frequent microorganism identified in some studies performed in the pediatric population. This microorganism, especially affecting infants and preschoolers, produces a slightly different clinical picture when compared to *S. aureus* and other pyogenic microorganisms, induces less fever and a more insidious picture, generally associated with respiratory viral infections. It is important to optimize its detection since this infection can be confused with a transient synovitis, additionally, it is resistant to clindamycin and vancomycin, which are the most commonly used empirical antibiotics in regions with high incidence (≥20%) of MRSA. Rapid detection enables the initiation of ampicillin/sulbactam or first or higher generation cephalosporins, which adequately cover it (3, 19–22). According to recent guidelines published by PIDS and IDSA, empirical management against *S. aureus* should be provided in patients with suspected osteoarticular infection (6, 23). Cefazolin provides adequate coverage against methicillin-sensitive *Staphylococcus aureus*, *Streptococcus pyogenes*, *Streptococcus pneumoniae*, and *Kingella kingae* (22). Empirical coverage against MRSA is recommended in regions with a methicillin resistance prevalence greater than 20% (6, 23), this and other studies conducted in Colombia demonstrate a high prevalence of CA-MRSA (38%–53%) and low resistance to clindamycin (7%) (14, 15). Information regarding the prevalence of *Kingella kingae* in our setting is scarce. We found *Kingella kingae* in 4% of patients, a lower rate than that reported in studies in the Northern Hemisphere (3, 19–21), similar to studies conducted in Chile (5, 21), therefore, in our setting, empirical management with clindamycin is recommended for stable patients and vancomycin for unstable patients*.* The implementation of molecular techniques in clinical practice in Latin America will allow us to know the real prevalence of this microorganism and explore why it has been found less often in South America than the Northern Hemisphere.

The panel also increased the detection of *Streptococcus pyogenes*, and other studies have identified it as a cause of BJI (3, 5, 17, 24). Detecting this microorganism is important due to the possibility of generating sepsis and toxic shock syndrome (24). In addition, its detection allows us to direct antibiotic therapy to crystalline penicillin or a first-generation cephalosporin, reducing the use of clindamycin or vancomycin and the adverse events that they can generate.

The correlation between the isolates in culture and the panels both in the microorganism with resistance found in the present study is similar to that found in previous studies carried out in adults and children using the same panel (17, 18, 25). The correlation between the culture and the molecular panel of the samples taken from bone secretion was very good (*κ* = 1), currently, regulatory entities only approve the panel for joint fluid samples, which limits the use of the molecular panel in bone fluid/pus samples. We consider that more studies should be done to determine the usefulness of the panel in bone secretion samples.

In the present study, the cases in which the isolation was documented in the culture and not in the panel (2 *S. aureus* and one *S. agalactiae*), may be because the molecular test was performed on a sample different from the sample in which the culture was processed. In an infection, generally, several samples of joint fluid and bone pus are taken, and the bacterial load is different in each one, which can affect both the results of the cultures and the results of the molecular tests. This fact could explain the false negatives of the panel regarding the culture, but it would not explain the cases in which the panel molecular is positive with a negative culture. In these cases, we consider that it is not appropriate to say that these are false positives, because these tests are more sensitive than culture.

The strengths of this study are that, to our knowledge, it is first study in Latin America and the second in the world that evaluate the performance of multiplex panels for BJI in the pediatric population; that the patients were prospectively included; and that the clinical characteristics of the patients were described, letting us correlate the results of culture and PCR to clinical and paraclinical characteristics. Its limitation is that the molecular tests were carried out 1–3 years after the sample was collected due to difficulty obtaining the reagents for processing during the COVID-19 pandemic. To mitigate the loss of genetic material, the samples were adequately preserved at −70  °C.

## Conclusion

There is a moderate level agreemeent (*κ* = 0.47) between the BIOFIRE® Joint Infection panel and cultures when used in samples from patients with bone and joint infections. There was a very good level of agreement between the panel and culture for the MRSA detection (*κ* = 1). Among their advantages are that they help identify microorganisms that are difficult to isolate in culture, such as *Kingella kingae*. They identify coinfections and detect early the presence of bacterial resistance mechanisms that allow the guidance of antibiotic therapy in a timely manner, which is useful, especially in unstable patients in areas with high prevalence of MRSA. These panels can also help with the differential diagnosis with noninfectious pathology. Their implementation should be advocated in programs for the prudent use of antibiotics through protocols that allow their use in scenarios where their utility may be greater, for example, in preschool-aged patients where *Kingella kingae* is a frequent microorganism, in critically ill patients where definitive treatment must be promptly initiated or in patients with previous negative cultures, among others.

## Data Availability

The original contributions presented in the study are included in the article/Supplementary Material, further inquiries can be directed to the corresponding author.
