# Supplementary material for: Correlation between the results of cultures and the molecular BIOFIRE® joint infection panel in a cohort of pediatric patients with bone and joint infections in Bogotá, Colombia
Source: Front Pediatr. 2024 Apr 24;12:1359736. doi: 10.3389/fped.2024.1359736 (PMC11076823; doi:10.3389/fped.2024.1359736)
Supplement: Supplementary file 1 [file Table1.pdf]

Table 1S. Initial symptoms and comorbidities (supplementary material)

Variable	n	%
Initial symptoms		
• Affected limb pain	32	100
• Fever	24	75
• Joint edema	24	75
• Joint erythema	24	75
• Limp	22	69
• Cough	8	25
• Rhinorrhea	4	12.5
