# Supplementary material for: Correlation between the results of cultures and the molecular BIOFIRE® joint infection panel in a cohort of pediatric patients with bone and joint infections in Bogotá, Colombia
Source: Front Pediatr. 2024 Apr 24;12:1359736. doi: 10.3389/fped.2024.1359736 (PMC11076823; doi:10.3389/fped.2024.1359736)
Supplement: Supplementary file 2 [file Table2.pdf]

Table 2S. Test results on admission and at the end of hospitalization (supplementary material).

Upon admission	Range	Average	End of hospitalization	Range	Average
Leukocytes * (cells/mL)	2.050-28.630	12.567	Leukocytes Ħ (cells/mL)	3.380-18.600	9.365
Neutrophils * (cells/mL)	490-18.672	8.741	Neutrophils Ħ (cells/mL)	260-13.570	4.655
Lymphocytes * (cells/mL)	280-7.263	2.579	Lymphocytes Ħ (cells/mL)	390-8.946	3.278
Platelets * (cells/mL)	16.000-843.000	368.953	Platelets Ħ (cells/mL)	10.550-1.222.000	594.495
Hemoglobin * (g/dL)	7.7-15.5	11.7	Hemoglobin Ħ (g/dL)	8.5-14.6	11.5
Hematocrit * (%)	21.3-46,3	34.7	Hematocrit Ħ (%)	25-42.3	34
CRP * (mg/L)	0.6-446.2	111.7	CRP * (mg/L)	1.4-104	31.4
ESR * (mm/h)	12-70	40.4	ESR * (mm/h)	15-62.8	44.2
