# Supplementary material for: Correlation between the results of cultures and the molecular BIOFIRE® joint infection panel in a cohort of pediatric patients with bone and joint infections in Bogotá, Colombia
Source: Front Pediatr. 2024 Apr 24;12:1359736. doi: 10.3389/fped.2024.1359736 (PMC11076823; doi:10.3389/fped.2024.1359736)
Supplement: Supplementary file 3 [file Table3.pdf]

Table 3S. Radiological findings by type of imaging used:

Simple radiography. n = 30 patients	n (%) 39 reported findings	Joint ultrasound n = 27 patients	n (%) 49 reported findings	Nuclear magnetic resonance n = 22	n (%) 43 reported findings
• Normal	10(26)	• Increased intra-articular fluid	15(31)	• Bone abscess	11(25.5)
• Soft tissue edema	9(22)	• Soft tissue edema	13(27)	• Muscle and soft tissue abscess	11(25.5)
• Increased joint space	7(16)	• Normal	4 (8)	• Increased intra-articular fluid	9(20.9)
• Fat line attenuation	2(5)	• Subperiosteal collection	3 (6)	• Soft tissue edema.	7(16.3)
• Bony lytic changes	2(5)	• Joint effusion	2(4)	• Inflammation and bone destruction.	4(9.3)
• Suprapatellar bursa strain	2(5)	• Synovial thickening	2(4)	• Intramedullary collection	2(4.7)
• Joint thinning	1(3)	• Periosteal destruction	2(4)	• Synovitis	1(2.3)
• New periosteal formation	1(3)	• Tenosynovitis	2(4)		
• Subcutaneous emphysema	1(3)	• Metaphyseal periosteal prominence	1(2)		
• Hemophilic arthropathy	1(3)	• Deep vein thrombosis	1(2)		
• Osteochondrosis (Osgood Schlatter)	1(3)	• Myositis	1(2)		
		• Intramuscular collection	1(2)		
		• Increased fluid from the bursa	1(2)		
		• Hemarthrosis	1(2)		
